# The Kidney–Gut Axis as a Novel Target for Nutritional Intervention to Counteract Chronic Kidney Disease Progression

**DOI:** 10.3390/metabo14010078

**Published:** 2024-01-22

**Authors:** Sandra Cabała, Małgorzata Ożgo, Agnieszka Herosimczyk

**Affiliations:** Department of Physiology, Cytobiology and Proteomics, Faculty of Biotechnology and Animal Husbandry, West Pomeranian University of Technology Szczecin, Klemensa Janickiego 29, 71-270 Szczecin, Poland; sandra.cabala@zut.edu.pl (S.C.); malgorzata.ozgo@zut.edu.pl (M.O.)

**Keywords:** chronic kidney disease, gut microbiota, gut–kidney axis, nutraceuticals, functional ingredient

## Abstract

A well-balanced diet is integral for overall health, aiding in managing key risk factors for kidney damage like hypertension while supplying necessary precursors for metabolite production. Dietary choices directly influence the composition and metabolic patterns of the gut microbiota, showing promise as therapeutic tools for addressing various health conditions, including chronic kidney diseases (CKD). CKD pathogenesis involves a decline in the glomerular filtration rate and the retention of nitrogen waste, fostering gut dysbiosis and the excessive production of bacterial metabolites. These metabolites act as uremic toxins, contributing to inflammation, oxidative stress, and tissue remodeling in the kidneys. Dietary interventions hold significance in reducing oxidative stress and inflammation, potentially slowing CKD progression. Functional ingredients, nutrients, and nephroprotective phytoconstituents could modulate inflammatory pathways or impact the gut mucosa. The “gut–kidney axis” underscores the impact of gut microbes and their metabolites on health and disease, with dysbiosis serving as a triggering event in several diseases, including CKD. This review provides a comprehensive overview, focusing on the gut–liver axis, and explores well-established bioactive substances as well as specific, less-known nutraceuticals showing promise in supporting kidney health and positively influencing CKD progression.

## 1. Introduction

A well-balanced diet undoubtedly helps maintain overall health, while also aiding in the management and reduction of primary risk factors for kidney damage, such as diabetes and hypertension. It also serves as a primary supplier of the precursors necessary for metabolite production. In practice, dietary choices shape the composition of gut microbiota (GM), since the nutrients may directly influence the GM, impacting both its structure and the metabolic patterns of its microbial inhabitants, and thus emerge as promising therapeutic tools for addressing a variety of health conditions, including kidney diseases. A large and growing body of evidence solidifies the link between the Western diet intake, characterized by the excessive consumption of fatty and processed meats, saturated fats, salt, and sugars, and a simultaneous deficiency in fresh fruits and vegetables, with the onset of numerous diseases, including chronic kidney disease (CKD) [1]. As recently reviewed by Dobrek [2], a critical aspect of CKD pathogenesis also involves the interplay between the progressive decline in glomerular filtration rate (GFR) and the retention of nitrogen metabolic waste products. This dynamic interaction contributes to the emergence of gut dysbiosis and an excessive production of bacterial metabolites, such as phenols, indoles, and amines. As a result, it increases intestinal wall permeability, allowing these substances to enter the bloodstream, where they act as uremic toxins, perpetuating inflammation and enhancing oxidative stress in kidney tissues, ultimately playing a crucial role in tissue remodeling [2]. Therefore, dietary interventions seem to be of significant importance for patients with CKD, as they can help reduce oxidative stress and inflammation, thereby potentially slowing down the progression of CKD [3]. In this context, functional ingredients and nutrients like fiber, prebiotics, probiotics, synbiotics, and fatty acids, along with nephroprotective phytoconstituents, may play a pivotal role. They can either modulate pro- and anti-inflammatory pathways or exert their effects at the gut mucosal level.

As discussed previously, the relationship between gut microbiota and chronic kidney diseases is referred to as the “gut–kidney axis” [4]. It is well-established that the intestinal microbiome, which comprises various microorganisms, such as bacteria, viruses, protozoa, and fungi, exerts a significant impact on the host during homeostasis and disease. Gut microbes and their metabolites provide significant health benefits to the host, which encompass the reinforcement of gut epithelial integrity, the provision of energy, protection against pathogens, and immunomodulation [4]. Therefore, it is believed that an altered microbial composition, a condition known as dysbiosis, acts as a triggering event in the progression of several diseases, including CKD [5]. In this review, we explore the intriguing role of the gut–liver axis in relation to kidney health and disease. Here, we also delve into exploring the potential beneficial impacts of specific functional ingredients, essential nutrients, and nutraceuticals—some of which have received less attention but show promise in promoting kidney health and potentially influencing the progression of CKD.

## 2. The Kidney–Gut Axis: A Potential Connection between Gut Dysbiosis and CKD

The kidney plays a critical role in maintaining plasma osmolarity by intricately regulating water, solute, and electrolyte levels in the bloodstream. Beyond this, they also maintain an acid-base balance, produce essential hormones, and participate in specific metabolic functions. Of notable significance, the kidneys are indispensable in excreting nitrogenous waste products, including urea, creatinine, and ammonia ions. Consequently, any substantial alterations in renal function lead to the accumulation of these waste products within the body [6]. It should be emphasized that the kidneys’ ability to perform their functions is predominantly determined during fetal development. Throughout this phase, the formation of nephrons occurs, and the final number that is established before birth becomes the lifelong kidney endowment. A literature analysis reveals considerable variability in the number of nephrons, observed in both humans [7] and various animals, such as mice [8], rats [9], pigs [10], and sheep [11]. According to Bhat and Manolescu [7], the number of nephrons in a healthy human kidney typically ranges from 0.3 to 1.3 million. The above laid the foundation for the widely accepted concept that the number of nephrons acquired in utero is a crucial factor for proper kidney function and a determinant of cardiovascular issues in adulthood [7]. This further implies that a low nephron number may influence susceptibility to various renal pathologies, potentially accelerating the onset of CKD, also known as chronic kidney failure. Furthermore, CKD is linked to a diverse array of risk factors, with diabetes, hypertension, and polycystic kidney disease being the most prevalent, while other contributing causes include glomerulonephritis, nephrolithiasis, genetic and environmental risk factors, lifestyle behaviors, as well as acute and viral infections [12,13,14,15,16,17].

Recent findings strongly indicate that the gut microbiota (GM) have emerged as a key player in CKD pathogenesis, and the interaction between GM and CKD is reciprocal. CKD can influence the composition of the gut microbiota, leading to gut dysbiosis. Conversely, dysbiosis in CKD patients may elevate uremic toxin levels, thereby contributing to the progression of CKD [18,19,20]. Microorganisms, including bacteria, yeasts, and viruses, residing within the gastrointestinal tract, collectively referred to as the GM, play a crucial role in maintaining the overall balance and well-being of the host, although they can also act as a potential source of disease [21]. For instance, Stanford et al. [22], in their systematic review, demonstrated that individuals with CKD, in contrast to healthy controls, exhibit a decreased abundance of the *Prevotellaceae* family and *Roseburia* genus, coupled with increased numbers of potential pathobionts from the *Enterobacteriaceae* and *Streptococcaceae* families, as well as the *Enterococcus* genus. As kidney stones are also a contributing factor in the development of CKD and its progression, Stanford et al. [22] also made an attempt to display the relationship between gut microbiota and nephrolithiasis. Consequently, these authors showed that, in adults with kidney stones, there was a significant reduction in *Bifidobacterium* and *Faecalibacterium* taxa, while *Bacteroides* was more highly abundant.

From the moment of birth, the establishment of the microbiome is an extremely dynamic process, characterized by continuous changes in its composition, which are greatly influenced by a wide range of external factors, especially during the early stages of life. Elements like the delivery method, dietary preferences, hygiene practices, and medication use, particularly antibiotics, all play a significant role in shaping the final composition and diversity of the gut microbiota [23]. During the first 2–3 years of human life, the gut microbiome starts to develop, eventually stabilizing into a configuration that closely resembles the typical microbial taxonomy found in adults [24]. However, it should be pointed out that the structure of the microbial communities in microbiota can vary among individuals and species, and notably, it can also fluctuate within the same individual [21]. The presence of bacteria in the gastrointestinal tract gradually increases from the stomach to the large intestine, where they reach their highest concentration and species diversity [25].

**Figure 1:**
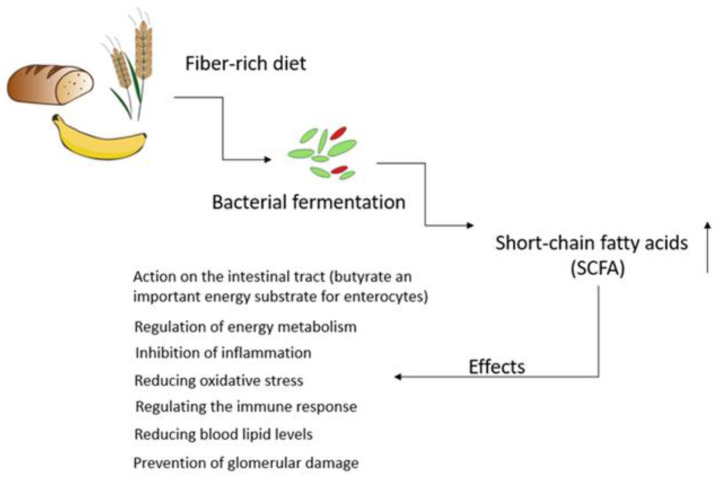
Effects of short-chain fatty acids on the body. SCFAs are the end products of the bacterial fermentation of complex polysaccharides. SCFAs are straight-chain saturated fatty acids, including acetate, propionate, and butyrate. SCFAs are involved in regulating energy metabolism, inhibiting inflammation and oxidative stress, and regulating the immune response.

This intestinal microbial species exert a remarkable influence on the absorption, metabolism, and storage of nutrients [4]. Most importantly, GM also play a critical role in facilitating the fermentation of a diverse array of compounds, especially those that that are resistant to digestion by human enzymes. This results in the generation of a diverse array of metabolites that can affect host cells, tissues, and organs [26]. Gut-microbiota-derived products encompass both intermediates and the end products of bacterial metabolic processes, and their ultimate composition in the gut is greatly influenced by the dietary intake of specific nutrients. The microbial fermentation of complex non-digestible dietary carbohydrates primarily occurs in the cecum and proximal regions of the colon. The large intestines also serve as a site for the fermentation of dietary proteins that escaped digestion in the upper regions of the gastrointestinal tract. Residual proteins and peptides are subjected to hydrolysis, breaking down into amino acids through the action of extracellular proteases and peptidases produced by intestinal microorganisms [27]. It should be emphasized that the non-digestible carbohydrate fermentation is much more favorable as it results in the production of short-chain fatty acids (SCFAs) and gases such as carbon dioxide, hydrogen, methane, and hydrogen sulfide [28,29]. SCFAs, particularly acetate, propionate, and butyrate, are recognized for their crucial roles in regulating the energy metabolism, preserving the integrity and functionality of the gut barrier, inhibiting inflammation and oxidative stress, and modulating the immune response (Figure 1) [30,31].

**Figure 2:**
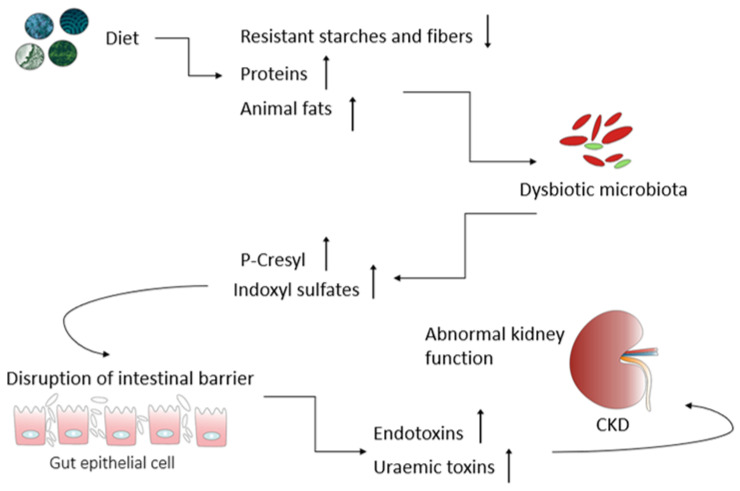
An inadequate diet contributes to the development of a dysbiotic intestinal microbiota. Intestinal dysbiosis leads to the increased production of potentially toxic metabolites such as indoxyl sulfate and p-cresyl sulfate, and increased permeability and damage to the intestinal barrier. Damage to the intestinal barrier results in increased host exposure to uremic toxins and endotoxins. The accumulation of uremic toxins in the body contributes to increased oxidative stress and inflammation, resulting in the development of chronic kidney disease (CKD).

On the other hand, undigested dietary proteins represent a significant source of nitrogen, urea, and other protein fermentation products in the colon. When there is inadequate dietary carbohydrate intake, a significant amount of α-amino nitrogen is generated from the microbial fermentation of aromatic amino acids such as tyrosine, phenylalanine, and tryptophan [32]. The alpha-amino nitrogen is subsequently metabolized in colonocytes and the liver to form so-called protein-bound uremic toxins such as p-cresyl sulfate (PCS), indoxyl sulfate (IS), indole acetic acid (IAA), kynurenate, phenyl sulfate, and uric acid. In patients with chronic kidney disease, the reduced renal excretion of PCS, IS, and IAA leads to an increased accumulation of these metabolites in the blood, and also in the intestinal epithelium [33]. The enhanced transfer of these gut-derived uremic toxins into the bloodstream can significantly elevate cardiovascular morbidity and mortality. This is due to their well-documented role in promoting vascular calcification through a variety of mechanisms, including the apoptosis of vascular smooth muscle cells, impairment of endothelial cell function, induction of oxidative stress, and interaction with the local renin-angiotensin–aldosterone system (Figure 2) [34].

**Figure 3:**
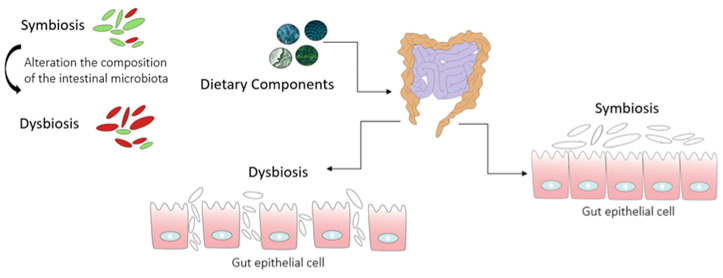
Abnormalities in the composition and function of the microbiota (intestinal dysbiosis) can damage the intestinal barrier, leading to an increased permeability.

A recent study conducted by Ondrussek-Sekac et al. [35] revealed that increased serum levels of IS were closely associated with podocyte dysfunction. This was evidenced by the reorganization of the actin cytoskeleton, which, in turn, led to a pro-inflammatory environment, resulting in reduced podocyte viability and impaired functionality. Moreover, in this modified intestinal environment (high urea and nitrogen concentrations), there is a subsequent promotion of the colonization of ureolytic bacteria. These microorganisms have the capacity to generate urease, which efficiently hydrolyzes urea, resulting in an increased production of ammonia. As a consequence, the elevated ammonia levels raise the gut pH, disrupt tight junctions, and foster endotoxemia and systemic inflammation [36]. Another uremic toxin, derived from the microbial metabolism of choline and L-carnitine, predominantly present in animal proteins such as red meat and eggs, is referred to as trimethylamine-N-oxide (TMAO) [37]. TMAO is considered as a potential promoter of atherogenesis as it enhances foam cell formation by elevating the expression of macrophage scavenger receptors, interfering with cholesterol and bile acid metabolism in the enterohepatic system, and hindering macrophage reverse cholesterol transport (RCT) [38]. Patients with CKD often exhibit alterations in the composition and functionality of the gut microbiota, a condition commonly referred to as gut dysbiosis [25]. This microbial population imbalance is characterized by an expansion of proteolytic bacteria and a significant decrease in saccharolytic ones, accompanied by a corresponding shift in the production of microbiota-derived metabolites. This, in turn, can lead to enhanced intestinal permeability (leaky gut syndrome), ultimately contributing to systemic inflammation (Figure 3) [39].

These findings are further supported by the results of a study conducted by Vaziri et al. [40], where a significant decrease in the expression of vital protein constituents of colonic tight junctions, including claudin-1, occludin, and ZO-1, was observed in a murine models of CKD. When the integrity of the intestinal tight junction barrier is compromised, luminal gut antigens, proinflammatory cytokines, and pathogen products can readily pass into the underlying intestinal tissue [32]. According to Vaziri et al. [40] this, in turn, prompts the activation of resident immune cells and the recruitment of inflammatory cells, resulting in the production of cytokines that further promote the influx of circulating inflammatory cells. The enhanced synthesis of these cytokines has been shown to be associated with the mucosal barrier disruption via the endocytosis and degradation of key tight junction proteins. Thus, the interplay between the impairment of the tight junction and inflammation creates a vicious cycle, boosting intestinal and systemic inflammation and gut barrier dysfunction [40]. As extensively reviewed by Lowenstein and Nigam [41], protein-bound uremic toxins play a crucial role in a broad remote sensing and signaling network, emphasizing the central role of transporter-mediated communication between organs and/or between the host and gut microbiota. Uremic toxins, upon entering the bloodstream, are transported to various organs, such as the liver, kidney, pancreas, brain, intestines and muscles, via solute carrier (SLC) and ATP-binding cassette (ABC) transporters. Within these organs, they are thought to induce gene expression changes and thus disrupt crucial signaling and metabolic pathways. Ultimately, these toxins are eliminated through the proximal tubule [42]. Key transporters, OAT1 and OAT3, expressed on the basolateral membrane of proximal renal tubules, are crucial for the renal uptake of endogenous metabolites, including uremic toxins, in exchange for α-ketoglutarate. Additionally, efflux pumps like BCRP and MRP2/4 concurrently transport these substances into the tubular lumen, facilitating their final removal [43,44]. In CKD patients, a decrease in transporters appears to be evident, as supported by the study of Naud et al. [45]. Their research demonstrated that adenine-induced chronic renal failure in rats affects the expression and activity of various kidney drug transporters, leading to intrarenal drug accumulation and reduced renal clearance. Considering the significant role of the gut–kidney axis in the development of chronic kidney disease, new treatment methods focus, among other aspects, on dietary factors that enhance the prevalence of anti-inflammatory and other health-promoting bacteria. This involves the use of probiotic, prebiotic, or synbiotic supplements and nephroprotective phytoconstituents to potentially delay or even reverse the disease progression [46].

## 3. Nutritional Strategies Focusing on the Gut Microbiota as a Novel Treatment for Counteracting CKD Progression

As recently reviewed by Naber and Purohitet [47], CKD is closely associated with several clinically important complications, including hypertension, hyperkalemia, hyperphosphatemia, and metabolic acidosis; however, these risks can be reduced, and the disease’s progression slowed, through the diligent monitoring of protein, phosphorus, potassium, sodium, and calcium levels in the diet, potentially alleviating the progressive loss of renal function symptoms. Given the close bidirectional association between gut microbiota and CKD progression, it is also crucial to emphasize the significance of gut-derived uremic toxins in this process. These toxins impact the intestinal, renal, and cardiovascular systems and are challenging to eliminate through standard dialysis methods. Therefore, they warrant special attention in the context of CKD treatment. The modulation of GM with an emphasis on increasing saccharolytic bacteria over proteolytic bacteria through the use of prebiotics, probiotics, and synbiotics could represent an especially promising potential therapeutic avenue.

Probiotics are living, non-pathogenic microorganisms that, when consumed in sufficient quantities, can inhabit the intestines and exert a positive influence on the gut microbiota by promoting the growth of beneficial bacteria [48]. These microorganisms can encompass a diverse range, including bacteria, yeast, and molds. Among them, the most commonly used probiotics include *Bifidobacteria*, *Lactobacillus*, *Propionibacteria*, *Bacillus*, *Akkermansia muciniphila*, and *Saccharomyces* [49]. *Lactobacillus-* and *Bifidobacterium*-based probiotics play a crucial role in altering gut pH, countering pathogens by generating antimicrobial compounds, and competing for binding sites with pathogens, as well as for available nutrients and growth factors, triggering immunomodulatory cells and producing enzymes like lactase, as well as essential vitamins such as B1, B4, B6, and B12 [50]. These probiotic strains also have the potential to restore the mucosal barrier of the gut and generate short-chain fatty acids through cross-feeding interactions, thereby promoting the growth of other colonic bacterial species. This process may help mitigate uremic toxicity, lower pro-inflammatory markers, and slow the progression of chronic kidney disease [51,52]. Additionally, the potential role of probiotics and entire microbial communities in therapeutic interventions has been explored in individuals with nephrolithiasis, who face twice the risk of reduced kidney function and CKD [53]. The identification of *Oxalobacter formigenes* (Oxf), an oxalate-degrading bacterium, has raised interest in its potential role in calcium oxalate stone disease, considering that approximately 75% of kidney stones are predominantly composed of calcium oxalate. Based on previous studies, a correlation appears to exist between the absence of this Gram-negative bacterium and the occurrence of hyperoxaluria, ultimately contributing to the formation of oxalate stones [54]. However, trials involving oxalate-degrading probiotics, such as those containing Oxf, *Lactobacillus*, and *Bifidobacterium*, have not produced definitive findings [55]. One of the major factors contributing to this limitation is the high sensitivity of *O. formigenes* to antibiotics and the specific conditions of probiotic formulation processes, including low-pH and low-oxygen conditions. Consequently, the potential utilization of this anaerobic bacteria for urolithiasis prevention is particularly restricted, especially in patients undergoing recent or current antibiotic therapy [56]. Additionally, it should be emphasized that there is no definitive and unequivocal answer regarding the optimal dosage, strain combination, and, most importantly, the duration for administering probiotics in patients with moderate to advanced CKD. Hence, variations in the effects can be observed across the interventional and clinical studies [57,58,59] available in the literature, all of which investigate the impact of dietary probiotic administration in patients with diverse CKD stages. These differences stem from variations in probiotic species, strains, dosages, and treatment durations (as detailed in Table 1).

On the other hand, prebiotics could provide an alternative to probiotics due to their well-documented, comprehensive, health-promoting effects, which may aid in mitigating chronic kidney failure [75]. The concept of prebiotics was initially introduced in 1995 by Gibson and Robefroid [76]. These authors defined prebiotics as dietary components that remain undigested in the upper gastrointestinal tract and have a beneficial influence on the host organism by selectively stimulating the growth and/or activity of bacterial populations in the colon [76]. In subsequent years (2004–2007), thanks to the simultaneous collection of data from both animals and humans, the concept of prebiotics expanded and was redefined as natural plant-derived ingredients that confer health benefits to the host by modulating the growth and activity of gut microbiota [77,78]. The current definition of prebiotics was introduced in 2016 during a meeting of the International Scientific Society for Probiotics and Prebiotics (ISAPP). The expert panel ultimately identified prebiotics as substrates that are selectively utilized by microorganisms, thus promoting improvements in host health [79]. Moreover, the definition of prebiotics has expanded to encompass non-carbohydrate compounds, including polyphenols and polyunsaturated fatty acids. Recent animal studies [80,81] have demonstrated the beneficial effects of these substances in protecting against chronic kidney disease and its associated complications. Long-chain polyunsaturated fatty acids, particularly n-3 polyunsaturated fatty acids (PUFA n-3), such as alpha-linolenic acid (ALA), eicosapentaenoic acid (EPA), and docosahexaenoic acid (DHA), have been demonstrated to exhibit antithrombotic, hypotriglyceridaemic, and anti-inflammatory effects [82]. Individuals with CKD have reduced levels of PUFA n-3 in their blood. This is due to factors such as the decreased intake of dietary sources rich in polyunsaturated fatty acids (e.g., fatty fish and fish oil), the presence of inflammation, impaired intestinal absorption, metabolic changes, and the loss of PUFA n-3 during dialysis. Therefore, dietary supplementation with n-3 PUFA, leveraging its dual effects of lowering blood pressure and mitigating inflammation, has the potential to slow down the progression of kidney diseases. This includes conditions such as immunoglobulin A nephropathy (IgAN), a primary cause of glomerulonephritis [83,84], as well as kidney disease progressing to end-stage renal disease (ESRD) [85]. Polyphenols, secondary metabolites synthesized by plants during development and in response to stress factors, constitute a group of water-soluble molecules that are primarily present in higher plants. This category includes flavonoids and phenolic acids, which act as natural antioxidants [86]. Recent research has extensively explored the impact of polyphenols on the gut microbiota. These compounds influence the composition of the gut microbiome by inhibiting pathogenic bacteria and stimulating beneficial bacteria. For example, resveratrol, a polyphenol synthesized by various plants, reaches the large intestine and interacts with the gut microbiome, leading to beneficial alterations in the microbial community [87]. Another noteworthy polyphenol is curcumin, found in turmeric. Its actions involve blocking the excessive production of lipopolysaccharides (LPS) and pro-inflammatory cytokines like interleukin-1 (IL-1) and tumor necrosis factor-alpha (TNF-α), and restoring gut barrier permeability [88]. According to Shen et al. [89], curcumin modulates gut flora by influencing the colonization of the members of the *Prevotellaceae*, *Bacteroidaceae*, and *Rikenellaceae* bacteria families.

As reviewed by Zirker [90], various types of prebiotic carbohydrates can be utilized in patients with CKD. These include inulin-type fructans (ITFs), which are divided into three subcategories: long-chain (inulin), medium-chain (oligofructose or fructooligosaccharides), and short-chain fructose monomers (short-chain fructooligosaccharides). Additionally, there are other prebiotic carbohydrates, like mannan oligosaccharides (MOS), xylooligosaccharides (XOS), galactooligosaccharides (GOS), raffinose oligosaccharide (RFO), isomaltooligosaccharide (IMO), and other non-starch polysaccharides (NSP), such as β-glucans, arabinoxylans, and pectins. Indeed, both dietary sources and prebiotic supplements are readily available to CKD patients [90]. Prebiotics naturally exist in various plant-based foods, including wheat bran, soy, raw potatoes, raw oats, unrefined wheat, wholegrain barley, onions, beans, green bananas, asparagus, and chicory [75,91]. They play a crucial role in CKD prevention by fostering the growth of health-promoting bacteria, such as *Bifidobacterium* and *Lactobacillus*. This, in turn, reduces the production of uremic substances originating in the colon while increasing the production of short-chain fatty acids [46]. For individuals with end-stage kidney disease who adhere to protein-restricted diets, prebiotics emerge as a crucial dietary supplement. They contribute to the elevation of short-chain fatty acids, positively influencing the metabolites produced by the gut bacteria and concurrently reducing systemic inflammation [52]. However, similar to probiotics, there is currently no established daily recommended dosage of dietary prebiotics for CKD patients. According to Douglas and Sanders [92], FOS can be supplemented at a concentration of 3 g per day, and mixed short- and long-chain inulin can be supplemented by up to 8 g daily. It is worth noting that several distinct factors can influence the effectiveness of carbohydrate-derived prebiotics in patients with chronic kidney disease. These factors include the mean degree of polymerization (the number of individual monosaccharide units in the polysaccharide chain), the dietary levels, the duration of intake, and the stage of CKD (Table 1).

Synbiotics are probiotic supplements combined with prebiotics, which have attracted significant interest in the context of chronic kidney disease therapy due to the potentially synergistic effects of their individual components (Table 1). However, the results obtained so far are inconclusive, and further research is necessary to confirm the efficacy of synbiotics as a therapy for chronic kidney disease [88]. For instance, a meta-analysis conducted by Yu et al. [52] assessed the effectiveness of dietary supplementation with probiotics, prebiotics, and synbiotics in individuals undergoing dialysis for end-stage renal disease. The findings revealed that prebiotics were particularly effective in reducing the plasma concentrations of interleukin-6 (IL-6) and tumor necrosis factor-alpha (TNF-α), as well as in lowering plasma levels of indoxyl sulfate (IS), malondialdehyde (MDA), and blood urea nitrogen (BUN). On the other hand, synbiotics demonstrated greater efficacy in lowering plasma levels of C-reactive protein (CRP) and endotoxins, including protein-bound uremic toxins. Similar results were observed when administering synbiotics, showing a reduction in plasma p-cresyl sulfate (PCS) levels, although not in the case of indoxyl sulfate (IS) [70,93]. The primary limitation of probiotic and synbiotic treatment is the lack of additional studies addressing the survival of probiotics in the already dysbiotic colon of CKD patients. When selecting probiotics, the participation of urease-containing bacteria should also be taken into account, as they increase ammonia production in the intestines, negatively affecting tight junctions and causing increased gut-barrier permeability, which, in turn, leads to the penetration of endotoxins from the intestinal lumen into the systemic circulation [25].

Medicinal plants have been used for centuries, with many modern drugs originating from natural products that were initially utilized in traditional medicine [94]. The demand for herbal remedies is increasing in both developing and developed countries due to their affordability and minimal side effects. Phytochemicals and medicinal plants are gaining significance in healthcare due to their natural origin and safety [95]. Plants and their extracts can be used as dietary supplements, herbal medicines, or as part of a healthy diet [96]. They contain organic compounds that exert specific physiological actions, including phenols, saponins, glycosides, flavonoids, alkaloids, tannins, steroids, and terpenoids [97]. Phytochemicals influence the gut microbiome by promoting the growth of probiotics and limiting the development of pathogens. Phytochemicals are distributed in various plant tissues, cell walls, and subcellular compartments, exhibiting diverse biological activities. These include antioxidant, chemopreventive, neuroprotective, cardioprotective, and immunomodulatory properties, as supported by research [98]. Importantly, they also exert nephroprotective effects, making them promising adjuncts in the treatment of kidney diseases, as they play a vital role in reducing oxidative stress, a crucial factor in the progression of CKD, which can induce glomerular and tubular damage. Additionally, oxidative stress is indirectly associated with inflammation, hypertension, and/or endothelial dysfunction [99]. In this respect, the most promising group acting as antioxidants are phenolic compounds that constitute the largest group of plant metabolites [100]. The main phenolic compounds include phenolic acids, tannins, stilbenes, lignans, and flavonoids. Flavonoids are synthesized by plants in response to microbial infection and exhibit antibacterial activity [101]. Flavonoids significantly impact kidney physiology and possess diuretic, natriuretic, and nephroprotective properties in cases of acute kidney injury (AKI) and CKD [102]. Major sources of flavonoids include herbs, grains, and citrus fruits, among others [103]. Alkaloids are secondary metabolites primarily composed of nitrogen and are utilized as bioactive components in medical applications. Tannins, on the other hand, have therapeutic properties, such as blood pressure reduction and bactericidal effects [104]. Anthocyanins are a subgroup of flavonoids responsible for the color of the fruits, leaves, and flowers of plants. Anthocyanins found in many fruits (blackberries, strawberries, and cherries) influence changes in gut microbiota composition. Most anthocyanins are not absorbed in the initial segment of the digestive system; they reach the colon, where they undergo biotransformation with the involvement of gut bacteria [105].

The identification and extraction of phytochemicals with potential therapeutic applications for kidney diseases can be achieved through screening studies. Worldwide, there is an ongoing exploration of plants and compounds that exhibit promise in the treatment of kidney disorders [106]. Table 2 provides a summary of potentially medicinal plants with a putative function regarding kidney functions.

## 4. Conclusions

In conclusion, the intricate relationship between the gut microbiota and overall health, particularly its impact on chronic kidney disease, underscores the importance of understanding and modulating this complex ecosystem. The gut microbiota’s crucial functions extend beyond nutrient absorption, metabolism, and storage to include the fermentation of non-digestible dietary carbohydrates, yielding short-chain fatty acids that play pivotal roles in energy metabolism, gut barrier integrity, inflammation inhibition, and immune response modulation. However, the delicate balance of the gut microbiota can be disrupted, especially in CKD, leading to the accumulation of uremic toxins, such as indoxyl sulfate, p-cresyl sulfate, and trimethylamine-N-oxide. These toxins contribute to cardiovascular morbidity and mortality, primarily through mechanisms like vascular calcification, inflammation, and oxidative stress. The dysbiosis observed in CKD, characterized by an imbalance between proteolytic and saccharolytic bacteria, results in enhanced intestinal permeability, systemic inflammation, and impaired tight junctions.

Novel therapeutic approaches focus on manipulating the gut–kidney axis through interventions targeting the gut microbiota. Probiotics, prebiotics, and synbiotics emerge as potential strategies. Probiotics, comprising beneficial microorganisms like *Lactobacillus* and *Bifidobacterium*, can positively influence the gut microbiota, but the optimal dosage and strain selection remain uncertain. Prebiotics, including dietary fibers and non-carbohydrate compounds, promote the growth of beneficial bacteria, potentially mitigating uremic toxicity and inflammation. Synbiotics, combining probiotics and prebiotics, present a synergistic approach with promising but inconclusive results in CKD therapy. Furthermore, medicinal plants and their phytochemicals offer a natural and diverse arsenal against kidney diseases. Phenolic compounds, alkaloids, tannins, and flavonoids, found in various plant sources, demonstrate antioxidant, anti-inflammatory, and nephroprotective properties. Their potential role in reducing oxidative stress and inflammation in CKD makes them attractive candidates for adjunctive therapeutic strategies.

In the quest to unravel the complexities of the gut–kidney axis, ongoing research will undoubtedly seek to identify optimal interventions, including dietary modifications and supplementation, to foster a healthy gut microbiota and mitigate the progression of chronic kidney disease. The multifaceted interactions within the gut microbiome and their consequences underscore the need for personalized and targeted therapeutic approaches tailored to the diverse landscape of individual microbiomes and CKD stages.

## Figures and Tables

**Table 1:** Selected studies involving probiotics, prebiotics, and symbiotics in patients with CKD. The arrows indicate the direction of changes (↑ enhancement, ↓ inhibition).

Study Design	Intervention	Results	References
Probiotic; randomized clinical trial; 42 hemodialysis patients; 4 weeks	1.6 × 10^7^ CFU/day of *L. Rhamnosus*	↓ Phenol and p-cresol serum levels	[60]
Probiotic; randomized, double-blind, placebo-controlled study; 46 outpatients with stage 3 and 4 CKD; 6-month	10^10^ CFU/day of a probiotic mix: *S. thermophilus*, *L. acidophilus*, and *B. longum*	↓ BUN No change in Cr and uric acid	[58]
Probiotic; randomized, double-blind, clinical trial; 70 hemodialysis patients; 3-month; one capsule a day	Gram-positive mix: *Lactobacillus Plantarum* A87, *Lactobacillus rhamnosus*, *Bifidobacterium bifidum* A218 and *Bifidobacterium longum* A101	↓ Syndecan-1 ↓ Blood glucose	[61]
Probiotic; randomized, double-blind, placebo-controlled study; low-protein diet; 60 patients; two doses daily for one month; one dose daily for another two months	5 × 10^9^ *Bifidobacterium longum*; 1 × 10^9^ *Lactobacillus reuteri*; maltodextrin	↓ Microflora toxins ↓ Blood urea nitrogen ↓ Total cholesterol ↓ Triglycerides	[62]
Prebiotic; randomized clinical trial; 32 patients with stage 3 and 4 CKD; non-dialysis; 8 weeks;	30 mm thrice/day of lactulose syrup	↓ Cr ↑ Bifidobacteria ↑ Lactobacillus	[63]
Prebiotic; chronic renal failure (CRF) cross-over method after randomization; 5 weeks	40 g/day fermentable carbohydrate (25 g wholemeal bread + 4.5 g inulin + 10.5 g crude potato starch)	↑ Nitrogen (N) in stool ↓ Nitrogen (N) excreted in the urine ↓ Plasma urea concentration	[64]
Prebiotic; randomized control trial, single-center, single-blind study; 59 patients with stage 3–5 CKD;	13.5 g of prebiotic fiber supplement with ß-glucan (GlucaChol-22^®,^, Bryanston, South Africa) daily	↓ Uremic toxins ↓ Total pCG and free pCG	[65]
Prebiotics; prospective, quasi-experimental single-center study; 30 hemodialysis patients; 8 weeks;	75 mg *Lactobacillus acidophilus* La-14 2 × 10^11^ CFU/g and 65 mg prebiotic fructooligosaccharides; once daily	↓ IS and p-CS in plasma ↓ IL-6	[66]
Synbiotics; randomized, double-blind, placebo-controlled; crossover study of symbiotic therapy; 37 patients; 6 weeks	SYNERGY; 15 g prebiotic, mix of 3 different types of fiber, probiotic 1 × 10^9^ (CFU) of 9 different strains of *Lactobacillus*, *Bifidobacteria* and *Streptococcus*; daily dose	↓ PCS in serum ↑ Bifidobacterium ↓ Ruminococcaceae	[67]
Prebiotics; double-blind, controlled study; 46 patients with CKD; 3 months	12 g FOS per day	↓ IL-6	[68]
Synbiotics; randomized, single-blind, placebo-controlled; 23 patients with stage 3b-4 CKD; 2 months	NATUREN G^®^(Canosa di Puglia, Italy) mix *Lactobacillus*, *Bifidobacteria*, FOS, inulin and natural antioxidants	↓ IS ↓ Small intestinal permeability, ↓ Abdominal pain and constipation syndromes	[69]
Synbiotics; randomized, double-blind, placebo-controlled trial; 30 patients in stages 3–4 of CKD; 4 weeks	Probinul neutro^®^ (Rome, Italy); 5 g three times a day; *Lactobacillus plantarum*; 5 × 10^9^ CFU *Lactobacillus casei* subsp. *Rhamnosus* and *gasseri* 2 × 10^9^ CFU *Bifidobacterium infantis* and *longum* 1 × 10^9^ CFU *Lactobacillus acidophilus*, *salivarius* and *sporogenes* 1 × 10^9^ CFU *Streptococcus thermophilus* 5 × 10^9^ CFU Prebiotic inulin 2.2 g and 1.3 g resistant starch	↓ Total plasma concentration of p-cresol	[70]
Polyphenol in cranberry; randomized, double-blind, placebo-controlled study; 25 patients with CKD; 2 months	500 mg of dry cranberry extract (2 times daily), and the placebo group received 500 mg of cornstarch (2 times daily)	No change in LPS and uremic toxins plasma levels	[71]
Trans-resveratrol; placebo-controlled crossover study; 20 nondialyzed patients with CKD; 16 weeks	500 mg trans-resveratrol (one capsule/day) for 8 weeks	↓ IS, ↓ p-CS ↓ IAA	[72]
Omega-3 fatty acids; prospective, randomized, double-blind study; patients with CKD undergoing hemodialysis; 12-week	4 capsules (2.4 g) of omega-3 fatty acids daily; placebo group 4 capsules of paraffin oil	↓ C-reactive protein ↓ IL-6 ↓ TNF-α,	[73]
Omega-3 fatty acids; randomized placebo-controlled trial; 73 nondiabetic patients with stage 3–4 CKD; 8 weeks	Omega-3 fatty acids 4 g daily	↓ IL-18 No change highly sensitive C-reactive protein and IL-12	[74]

**Table 2:** Summary of potentially medicinal plants with a putative function regarding kidney functions. The arrows indicate the direction of changes (↑ enhancement, ↓ inhibition).

Plant	Study Design	Research Results	References
*Salacia chinensis*	Pilot study; 30 stable diabetic CKD patients; *Salacia chinensis* 1000 mg twice daily	↓ homocysteine ↓ IL-6	[107]
*Hygrophila spinosa*	Analysis of the phytochemical profile, bactericidal activity of *Hygrophila spinosa* against multidrug-resistant *Pandoraea sputorum*, and examination of their hepatoprotective and nephroprotective activities on HepG2 and HEK 293 cell lines.	Methanol extract shows hepato- and nephroprotective effects against CCl_4_ and cisplatin induced cytotoxicity on HepG2 and HEK 293 cell lines, respectively. Bactericidal efficacy of phytohormones against multidrug-resistant *P. sputorum* was demonstrated.	[108]
*Potentilla anserine* L.	Investigating the protective effect of rosamultin against cisplatin-induced nephrotoxicity.	↓ BUN ↑ In vitro viability of HEK293 cells Inhibition of cisplatin-induced apoptosis, in vivo amelioration of renal dysfunction, and reduction in renal tubular damage.	[109]
*Glycyrrhiza glabra* L.	Phytochemical analysis and investigation of the nephroprotective potential of *Glycyrrhiza glabra* L. root extract against cisplatin in vitro and in vivo.	The nephroprotective effect of *G. glabra* roots can be attributed to antioxidant, anti-inflammatory and anti-apoptotic activities. Therefore, it has promising potential in the treatment of nephrotoxicity.	[110]
*Sida cordata*	Evaluation of antioxidant activity against CCL_4_-induced nephrotoxicity and analysis of phytochemicals of ethyl acetate from *Sida cordata*	The results indicate a protective role for ethyl acetate from *S.cordat* against CCl4-induced nephrotoxicity in rats, which is related to the antioxidant compounds it contains.	[111]
*Smilax cordifolia* *Eryngium carlinae*	To evaluate the effects of decoction of two plants, *Smilax cordifolia* and *Eryngium carlinae,* on renal dysfunction in rats.	Herbal decoctions decreased serum uric acid, albumin, and urea concentrations, accumulation of proteins associated with the formation of glomerular sclerosis and renal tubular fibrosis, increased creatinine clearance and concentration of pro-inflammatory and protective proteins	[112]

## Data Availability

Not applicable.
